# Excessive Biologic Response to IFNβ Is Associated with Poor Treatment Response in Patients with Multiple Sclerosis

**DOI:** 10.1371/journal.pone.0019262

**Published:** 2011-05-13

**Authors:** Richard A. Rudick, M. R. Sandhya Rani, Yaomin Xu, Jar-Chi Lee, Jie Na, Jennifer Shrock, Anupama Josyula, Elizabeth Fisher, Richard M. Ransohoff

**Affiliations:** 1 Mellen Center for Multiple Sclerosis, Neurological Institute, Cleveland Clinic, Cleveland, Ohio, United States of America; 2 Department of Neurosciences, Neuroinflammation Research Center, Lerner Research Institute, Cleveland Clinic, Cleveland, Ohio, United States of America; 3 Quantitative Health Sciences, Lerner Research Institute, Cleveland Clinic, Cleveland, Ohio, United States of America; 4 Department of Biomedical Engineering, Lerner Research Institute, Cleveland Clinic, Cleveland, Ohio, United States of America; National Institutes of Health, United States of America

## Abstract

**Background:**

Interferon-beta (IFNβ) is used to inhibit disease activity in multiple sclerosis (MS), but its mechanisms of action are incompletely understood, individual treatment response varies, and biological markers predicting response to treatment have yet to be identified.

**Methods:**

**T**he relationship between the molecular response to IFNβ and treatment response was determined in 85 patients using a longitudinal design in which treatment effect was categorized by brain magnetic resonance imaging as good (n = 70) or poor response (n = 15). Molecular response was quantified using a customized cDNA macroarray assay for 166 IFN-regulated genes (IRGs).

**Results:**

The molecular response to IFNβ differed significantly between patients in the pattern and number of regulated genes. The molecular response was strikingly stable for individuals for as long as 24 months, however, suggesting an individual ‘IFN response fingerprint’. Unexpectedly, patients with poor response showed an exaggerated molecular response. IRG induction ratios demonstrated an exaggerated molecular response at both the first and 6-month IFNβ injections.

**Conclusion:**

MS patients exhibit individually unique but temporally stable biological responses to IFNβ. Poor treatment response is not explained by the duration of biological effects or the specific genes induced. Rather, individuals with poor treatment response have a generally exaggerated biological response to type 1 IFN injections. We hypothesize that the molecular response to type I IFN identifies a pathogenetically distinct subset of MS patients whose disease is driven in part by innate immunity. The findings suggest a strategy for biologically based, rational use of IFNβ for individual MS patients.

## Introduction

Interferon beta (IFNβ) is routinely used to treat multiple sclerosis (MS), and in randomized placebo-controlled trials, reduced relapse rates by 30% [1]–[3]. However, clinical response varied among individuals, and *post-hoc* analyses of one study revealed that about 20% of IFNβ recipients were poor responders (PR), defined as ≥3 new T2 hyperintense brain lesions occurring within 2 years of treatment onset [1], [4]. Other studies by independent groups [5]–[7] consistently demonstrate that new brain MRI lesions develop in 20% to 25% of patients during IFNβ treatment and predict an unfavorable clinical outcome. Why some patients respond poorly to IFNβ treatment, whereas others respond better is not understood, and there are no validated biomarkers that predict treatment response for individual patients.

IFNβ (a type I IFN) treatment is therapeutic in MS patients, as indicated by clinical trial patient group results. However, results from a clinical trial of interferon gamma (IFNγ) (a type II IFN) [8], [9] demonstrated disease activation in some patients. Because type I and II IFNs regulate overlapping sets of IFN-regulated genes (IRGs), it is possible that some patients may worsen with IFNβ treatment. Such an outcome would not be evident from controlled clinical trials, where the majority of patients improve with treatment. Type I IFN is a cardinal mediator of innate immunity, whereas type II IFN participates in both innate and adaptive immunity. In the IFNγ trial, MS disease activation was interpreted as implicating T_H_1-mediated processes in MS pathogenesis. Development of type I IFN therapy continued, however, leading to IFNβ approval.

Increased bioavailability of type I IFNs is involved in diverse autoimmune diseases [10], and increased expression of type I IRGs has been detected in a subset of untreated MS patients [11], suggesting that some patients have augmented innate immunity, which is detected by monitoring type I IFN IRG expression in the absence of IFNβ injections. Moreover, several neuropathology and immunology studies of IFNβ treatment suggest that MS immunopathogenesis may differ between patients [12], [13]. Therefore, it is plausible that most MS patients improve with IFNβ therapy, while a minority worsen.

Poor clinical response to IFNβ can be related to production of IFNβ-neutralizing antibodies or to genetic variants in IFNβ receptors or signalling components [14]. In both categories, patients have reduced IFNβ bioavailability. However, such patients account for a minority of PRs [15]. In the remaining patients, poor response to IFNβ might relate to the nature of the IFNβ response, a relationship that could yield insight to the pathogenesis of MS, as well as strategies to personalize the use of IFNβ. Microarray-based cross-sectional expression analyses and studies of individual candidate genes support this concept [16], [17]. Here, we addressed the general hypothesis that the molecular response to IFNβ correlates with treatment response in individual patients with MS, and thus might provide biological markers useful to estimate prognosis, guide therapy, or offer insights into pathogenesis.

## Methods

### Clinical Protocol

The Cleveland Clinic Institutional Review Board approved the study. After discussion with our bioinformatics colleagues, but without a formal power calculation, we sought to enroll 100 patients. All subjects provided written informed consent. Subjects were eligible if they had clinically isolated syndrome (CIS) or relapsing-remitting MS, were treatment-naïve, were initiating intramuscular IFNβ-1a treatment, and were followed at the Mellen Center (our MS center). Each patient was examined at baseline, 6, 12, and 24 months. At 3 and 18 months, patients were contacted by phone to assess treatment compliance and ascertain side effects. At baseline, 6, and 24 months, blood was collected for IRG analysis in a clinical research unit immediately before and exactly 12 hours after an IFNβ injection, and the patients had standardized brain MRI scans for quantitative assessment of lesions and brain atrophy. At each visit, patients had neurological exams to determine the Kurtzke Expanded Disability Scale Score [18], the Multiple Sclerosis Functional Composite score [19], and history of intercurrent relapses or illness; they also were given a structured questionnaire to characterize flu-like symptoms, muscle aches, chills, fatigue, headache, and loss of strength. Serum was tested for IFN-neutralizing antibodies at 6 and 24 months. This report focuses on the 85 subjects with complete data at baseline and 6 months.

### MRI Analysis

The MRI acquisition included T2-weighted fluid-attenuated inversion recovery (FLAIR), T2- and proton density-weighted dual echo fast spin echo, and T1-weighted spin echo images acquired before and after injection of a standard dose of gadolinium (0.1 mmol/kg). Images were analyzed using software developed in-house to determine brain parenchymal fraction (BPF), T2 lesion volume, gadolinium-enhancing lesion volume and number, and the numbers of new and enlarging T2 lesions. BPF was calculated from FLAIR images using fully automated segmentation software [20]. Details of the lesion analysis methodology have been previously described [21]. Briefly, T2 hyperintense lesions were automatically segmented in the FLAIR and T2/PD images and visually verified using interactive software to correct misclassifications. Six-month follow-up images were registered to baseline, and intensity was normalized. Baseline T2 lesion masks were applied to the registered 6-month images to identify persistent lesions. The baseline images were then subtracted from the registered, intensity-normalized 6-month images to automatically identify new and enlarging T2 lesions at 6 months. For detecting enlarging lesions, the registration and subtraction software has cut-offs based on scan-rescan reproducibility of individual lesion volumes. For lesions ≥ 150 mm^3^, the lesion must grow by 20% to be counted as enlarging. For lesions <150 mm^3^, the lesion must grow by 50% to be counted as enlarging. New and enlarging T2 lesions were visually verified using interactive software to generate the final counts.

### RNA isolation

RNA was extracted *ex-vivo* from blood using the PAXgene RNA blood extraction kit (PreAnalytix, Switzerland) per the manufacturer's instructions and concentrated by ethanol precipitation. RNA quality and quantity were assessed by spectrophotometry (absorbance ratios, 260/280 nm) and visualized by agarose gel electrophoresis. Samples were stored at −80°C.

### Genes analyzed by macroarray

The cDNA macroarray analysis was performed as previously described [22], [23]. IRGs on a custom macroarray were represented by 166 human cDNAs selected from the Unigene database. Table S1 lists all genes on the macroarray with their GenBank accession numbers. These type I IFN IRGs were identified by microarray analysis of fibrosarcoma, epithelial, or endothelial cell lines treated with IFN-α or IFNβ [22], [23]. All were known IRGs.

The protocol for spotting DNA on the membrane, probe labeling, and hybridization has been described previously, with modifications as follows [22], [23]. Total RNA, 5 µg, isolated *ex-vivo* from blood was used for generating radiolabeled cDNA probes by reverse transcription with Superscript II in the presence of ^32^P-dCTP (Invitrogen, Carlsbad, CA). Residual RNA was hydrolyzed by alkaline treatment at 70°C for 20 min after which cDNA was purified using G50 columns (GE Healthcare, Buckinghamshire, UK). Preparation of macroarrays and hybridization of radioactive cDNA were conducted as described previously [22], [23]. Induction ratios (IRs) were calculated from radioactivity bound to the membranes.

To minimize variability, each patient's samples at baseline and 6 months were processed in a single batch experiment. A detailed laboratory protocol for the macroarray method is available on request.

IRs were validated using real-time (rt) quantitative PCR for 5 genes: OASL (accession number NM003733); TRAIL (U37518); IFI44 (D28915); HLADRA (J00194); and TIMP-1 (M59906). Spearman correlation coefficients between IRs calculated from rt-PCR and macroarray data for OASL, TRAIL, IFI44, HLADRA, and TIMP-1 were 0.92, 0.75, 0.36, 0.72, and 0.54, respectively, all statistically significant.

### Statistical analysis

Poor response to IFNβ was based on quantitative MRI analysis, comparing the MRI at the 6-month visit with baseline. Poor response was defined as the occurrence of ≥3 new or enlarging lesions. Differences in baseline characteristics between good responder (GR) and PR groups were compared using t-tests or Fisher's exact tests, as appropriate. A Poisson regression was used to test group differences in the number of induced IRGs with IRs≥2.0 at the baseline injection. Pearson correlation coefficients of log2 transformed IRs at initial injection compared with 6 months were computed for all 85 patients. Baseline, 6-month, and 24-month pair-wise correlations were computed for 5 randomly selected patients from each group.

To minimize noise, a filter was applied to exclude IRGs that were not regulated in this study. Genes with mean IRs within the range of 0.9 to 1.1 (i.e., +/- 10% from an IR of 1.0, or no change) were excluded. Using this method, 48 of the 166 IRGs were eliminated from further analysis at the initial IFNβ injection, and 30 IRGs were eliminated at the 6-month injection. For the remaining IRGs, covariate-adjusted least-square means (LS means) of the log2-transformed IRs were computed and compared between response groups by ANCOVA. The covariates were baseline age, sex, presence of gadolinium-enhancing lesions, and T2 volume.

The remaining IRGs were classified as upregulated or downregulated as follows: an IRG was upregulated if more than 50% of the 85 subjects had an IR>1.0; an IRG was classified as downregulated if more than 50% had an IR<1.0. For each IRG, LS means of the IRs were compared between PRs and GRs. The response was classified as exaggerated at the group level if PRs>GRs in upregulated genes, or PRs<GRs in downregulated genes. The proportion of genes showing an exaggerated response in PRs was tested (one-sided) with a binomial proportion test assuming a null hypothesis of proportion≤0.5.

To further investigate whether IRGs could discriminate between the responder groups, the magnitude of the exaggeration in each patient was computed as a sum of the exaggerated amounts (absolute differences between individual IRs and IR medians) for each regulated IRG. That is, for upregulated genes, if the individual IR was greater than the median for the whole group, or for downregulated genes, if the individual IR was less than the median for the whole group, then the exaggerated amount was calculated. The magnitudes of exaggerated responses were compared in PRs and GRs at the first and 6-month injections using ANCOVA with baseline demographic features, presence of baseline gadolinium lesions, and baseline T2 lesion volume as covariates.

## Results

### Research Subjects

Of the 99 subjects enrolled, 85 continued to take intramuscular IFNβ-1a for at least 6 months, which was the predetermined time-point for determining treatment response based on MRI, for correlation with IRG macroarray results. Reasons for drop out included: side effects (n = 4); new health issues (n = 2); no explanation provided (n = 4); left area (n = 1); switched to glatiramer acetate per patient preference (n = 1); sample hybridization not adequate at baseline or 6 month injection (n = 2). Subjects were encouraged to remain in the protocol for 24 months, so that the relationship between 6-month MRI activity and later disease progression, as well as stability of the IFNβ molecular response, could also be determined. At the time of this report, subject accrual was complete, and all subjects had 6-month visits. This report focuses on the analysis of 85 patients with complete data at baseline and 6 months. Of the 14 patients who were enrolled in the study but did not complete the planned 6-month macroarray analysis, 12 discontinued IFNβ-1a, whereas sample hybridization was unsuccessful in the other 2, either at first injection or 6 months. Ten patients who had completed the 24-month protocol contributed blood for 24-month IRG analysis. Baseline demographic and disease characteristics did not significantly differ between those who completed the first 6 months and those who did not (data not shown). For all other analyses, only the 85 patients who completed the first 6 months were included. Among these 85, 32% had CIS with multiple brain MRI lesions, and 68% had relapsing-remitting MS. The mean age was 35.7 years; mean disease duration was 2.4 years; 65% were women; and 91% were white. At 6 months, 15 (18%) subjects were classified as PRs based on our pre-determined MRI definition. The two groups were similar at baseline on all characteristics except that a higher proportion of PRs had gadolinium-enhancing lesions and greater T2 lesion volumes at baseline, indicating PRs had more severe disease **(** Table 1 **)**.

**Table 1:** Comparison of baseline characteristics between patients with a good vs poor response to IFNβ treatment.*

Characteristic	Good Responders (n = 70)	Poor Responders (n = 15)	All Patients (n = 85)	P-value GR vs PR
Age (years)	36.3 (9.4)	33.0 (11.2)	35.7 (9.8)	0.30
Symptom duration (years)	2.5 (3.0)	1.2 (1.7)	2.4 (2.9)	0.39
Female (%)	69	47	65	0.11
White (%)	93	80	91	0.14
CIS /RRMS (%/%)	34 / 66	20 / 80	32 / 68	0.37
EDSS	1.6 (1.0)	1.6 (1.2)	1.6 (1.0)	0.91
MSFC score	0.39 (0.48)	0.19 (0.41)	0.35 (0.47)	0.10
Patients (%) with gad-enhancing lesions	24.3	53.3	29.4	0.03
Gad-enhancing lesion volume (mm^3^)	0.097(0.38)	0.44 (0.72)	0.16 (0.47)	0.09
T2 Volume	3.0 (3.7)	5.8 (3.9)	3.5 (3.8)	0.02
T1 BH Volume	0.55 (0.75)	0.87 (0.82)	0.61 (0.77)	0.19
BPF	0.858 (0.014)	0.859 (0.013)	0.859 (0.014)	0.79

*All values are mean±SD, unless otherwise indicated.

CIS = clinically isolated syndrome; RRMS = relapsing-remitting multiple sclerosis; EDSS = Expanded Disability Scale Score; MSFC = Multiple Sclerosis Functional Composite; Gad = gadolinium; BH = black hole; BPF = brain parenchymal fraction.

### IRG Response to First Injection and Stability over Time

Preliminary assays in healthy subjects not receiving IFNβ showed that IRs did not vary more than 1.5-fold in assays separated by 12 or 24 hours. Therefore, an IR≥2.0 defined induction of an IRG. The number of induced IRGs at the first IFNβ injection varied among patients, ranging from 7 to 135, with no relationship between IFNβ responder status and number of induced genes (p = 0.76) (data not shown). Similarly, the pattern of response to the initial IFNβ injection varied considerably between patients as previously reported^22^.

**Figure 1:**
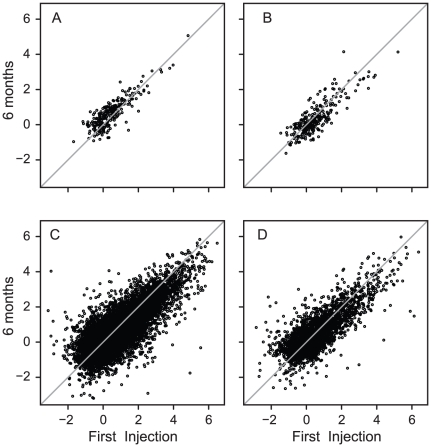
Correlations between induction ratios (IRs) for two patients at the initial (baseline) and 6-month injections. A) Patient 2 had a good treatment response; B) Patient 1 had a poor treatment response; C) All genes in all patients with a good treatment response; and D) All genes in all patients with a poor treatment response. Identical IRs at the two time points fall on the diagonal line whereas deviation from the diagonal reflects changes in the IRs at the first injection compared with the 6-month injection. Note the highly conserved IRG response at the two time points, both at the initial injection and 6 months later. There were no differences in consistency of the response in the good vs poor responders. Supplemental Figure 1 shows correlations for each of the 85 patients, including patients 1 and 2.

Figure 1 shows the IRs at first injection plotted against IRs at 6 months for a representative patient with a good response (Fig 1A), a representative patient with a poor response (Fig 1B), for all 70 patients with a good response (Fig 1C) and for all 15 patients with a poor response (Fig 1D). Despite inter-individual variability in the pattern and magnitude of IRG response after the first IFNβ-1a injection, the response was remarkably stable over time for individual subjects. Figure S1 shows the IRs at baseline and 6 months for each of the 85 patients. Two subjects (patients 7 and 25) had viral infections at the initial injection and so had little or no IRG induction at the first injection due to high pre-injection IRG expression levels. Both subjects responded to IFNβ injection at 6 months. Patient 21 developed neutralizing antibodies to IFNβ detected at 6 months. This subject responded briskly to the first IFNβ injection, but minimally at 6 months. Neutralizing antibody testing of all other subjects was negative at 6 months.

Excluding those three subjects, IRs at first injection strongly correlated with IRs at 6 months for individual patients [Pearson correlation coefficient mean (±SD) = 0.81±0.11]. The mean correlation coefficient for the 15 PRs (study numbers 1, 4, 12, 14, 18, 40, 49, 57, 62, 65, 66, 70, 87, 91, and 92) was 0.81±0.10, compared with a mean of 0.81±0.11 for the 67 GR patients (excluding subjects 7, 21, and 25, Figure S1).

**Figure 2:**
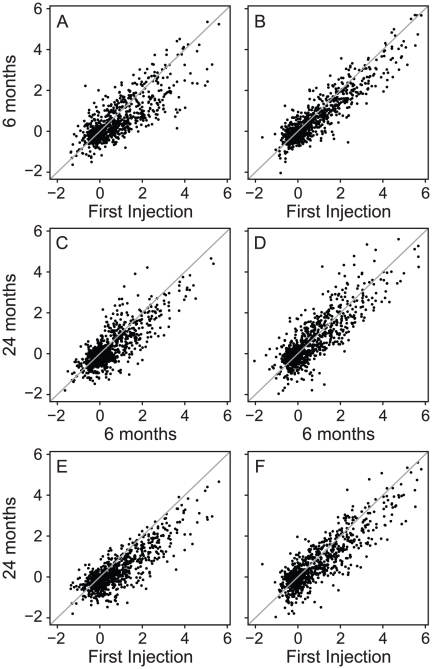
Scatter plots showing correlations between induction ratios between 3 time points over 24 months. Panels (A), (C) and (E): 5 good responders showing the correlation between the initial injection and the 6-month injection (A); between the 6-month and 24-month injection (C); and between the initial and the 24-month injection (E). Panels (B), (D), (F): 5 poor responders at the same time points. Note the highly conserved IRG response over 24 months, both in the good and poor responders.

The IRG analysis was repeated at 24 months for 10 selected patients (5 PRs and 5 GRs) **(** Figure 2 **)**. The IRG response was consistent over 2 years, with no appreciable difference between GRs and PRs. The IRs strongly correlated between baseline and 6 months (r = 0.80 for GRs, r = 0.91 for PRs); between 6 months and 24 months (r = 0.81 for GRs, r = 0.83 for PRs); and between baseline and 24 months (r = 0.83 for GRs, r = 0.87 for PRs). Figure S2 shows scatter plots for each patient, demonstrating highly consistent molecular responses to IFNβ injections over 24 months, regardless of responder status. These results suggested that treatment response status could not be attributed to attenuation of the molecular response to IFNβ over time.

### IRG Response in Good vs Poor IFNβ Responders

**Figure 3:**
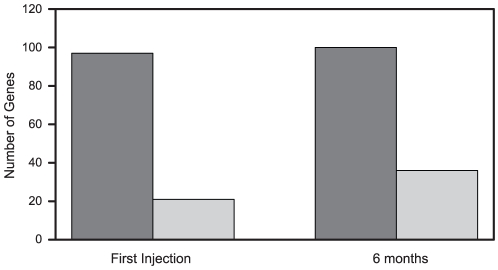
Number of genes with an exaggerated response in poor responders at the initial (A) and 6-month injection (B). The dark gray bars represent genes with an exaggerated response, and the light gray bars represent genes with no exaggerated response, as defined in the Methods section. The proportion of genes with an exaggerated response in PRs was significantly higher than 50% at first injection (p<0.001) and 6 months (p<0.001).

**Figure 4:**
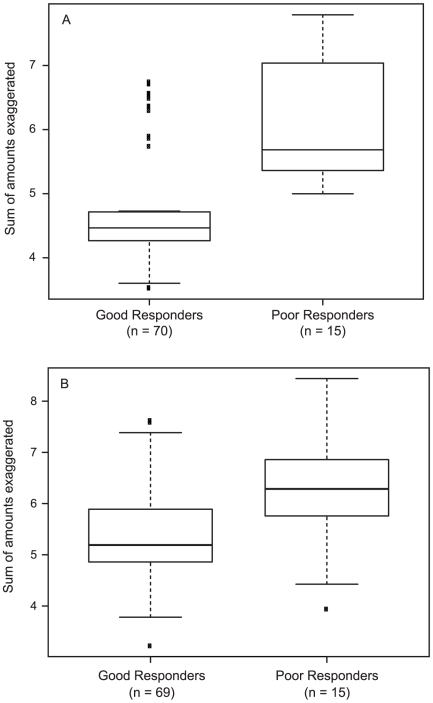
The magnitude of the exaggerated response for good and poor responder groups at the initial (A) and the 6-month (B) injection. The magnitude of exaggeration was significantly higher in PRs at the first (p = 0.007) and 6-month injections (p = 0.02).

The biological effects of IFNβ are traditionally accounted for by the activities of the IRG protein products [24]. We addressed whether the characteristics of the molecular response to IFNβ might explain PR status, either by revealing induction of deleterious inflammatory gene products [17] or selective failure of expression of beneficial genes [25]. In univariate analyses of the genes remaining after filtering, the covariate-adjusted LS mean IRs indicated differential responses (p≤0.05) between the PR and GR groups for 13 genes at the initial IFNβ injection, and 16 genes at the 6-month injection (Table 2). Unexpectedly, for all 13 genes at the initial injection, and for all 16 genes at the 6-month injection, the response, either induction or repression, was greater for patients with a poor response. This suggested an exaggerated IFNβ molecular response in the patients with a poor clinical response to IFNβ treatment. Figure 3 shows that 97 (82%) of 118 genes with mean IRs outside the 0.9–1.1 range showed an exaggerated response to IFNβ at the first injection, and 100 (74%) of 136 genes with mean IRs outside the 0.9–1.1 range showed an exaggerated response at the 6-month injection. The proportion of genes with an exaggerated response in PRs was significantly higher than 50% (p<0.001 at first injection and 6 months). Figure 4 shows the magnitude of the difference between the biological response for GRs and PRs at the first injection (figure 4A) and at 6 months (Figure 4B). the magnitude of exaggeration was significantly higher in PRs at the first injection (p = 0.007 and 6-month injection (p = 0.02).

**Table 2:** Univariate analysis with adjustment for covariates comparing induction ratios between good and poor responders to IFNβ.

Time of IFNβ Injection	Gene	Mean Adjusted IR		P Value
		Good Response	Poor Response	
Month 0	IL2	0.89	0.47	0.001
(n = 85)	ISG15-L	2.01	3.07	0.004
	TIMP-1	0.96	0.65	0.005
	IDO	3.18	5.17	0.008
	CD3e	0.73	0.54	0.012
	IFIT5	1.91	2.51	0.013
	FK506	1.29	1.65	0.013
	P4Ha1	1.14	1.41	0.020
	PI3K	1.50	1.99	0.026
	OASL	3.41	4.63	0.030
	HLADP	0.91	0.72	0.039
	TRAIL	4.50	6.23	0.048
	MT1X	2.95	4.53	0.049
Month 6	IFIT5	1.53	2.25	0.001
(n = 84)*	Elastase2	1.16	1.45	0.003
	CCR5	1.09	1.40	0.004
	UBE2L6	1.56	2.09	0.011
	Hou	1.54	2.35	0.016
	TRAIL	3.51	4.93	0.028
	TNFAIP6	2.17	3.05	0.029
	IL 15	1.28	1.64	0.030
	TOR1B	1.96	2.57	0.033
	IP-10	1.20	1.49	0.039
	Caspase 1	1.36	1.80	0.039
	FLJ20035	1.67	2.19	0.040
	PLSCR1	1.22	1.46	0.040
	IFN-44	2.00	2.68	0.043
	CEACAM	1.52	1.96	0.045
	RIG-1	3.27	4.60	0.045

IR = Induction ratio.

*1 patient with NAB with no biological response at 6 months was excluded.

## Discussion

Although IFNβ is the most commonly used disease-modifying treatment for MS, its mechanisms of action are not fully understood, no biological markers are available to guide individualized therapy, and differential clinical responsiveness is not understood at a mechanistic level. We sought to determine molecular correlates of the treatment response to IFNβ therapy in relapsing-remitting MS. Response to IFNβ was defined in advance, using MRI to classify treatment response in individual patients. In that regard, numerous reports have documented that patients developing new lesions while on IFNβ have relatively poor clinical outcomes. [4]–[7] Neutralizing antibodies were not observed at 6 months in any of the 15 patients classified as poor responders, and so could not have explained the poor response to therapy. We believe these 15 patients will have a poor clinical outcome compared with the patients classified as a good treatment response, but that differences will become evident only with longer follow-up. We continue to follow these patients, and will report outcomes in a future publication after all patients have been followed at least 2 years.

We initially hypothesized that 1) the molecular response to IFNβ would differ between patients but would be stable over time within individuals, and 2) treatment response would correlate with the nature of the primary molecular response or its persistence over time. We confirmed that the molecular response differs significantly between patients in regard to the identity and number of regulated IRGs and the extent of induction or repression.[22] Our study documents that the IRG response was remarkably consistent in individual MS patients for as long as 24 months, suggesting an individual IRG ‘fingerprint’. Exceptions to this finding (patients 7, 21, 25) were uncommon and readily explained. The molecular response remained consistent over time regardless of MRI outcome, excluding the possibility that attenuated IRG regulation explained the variable response to IFNβ. The groups differed, however, in the magnitude of IRG induction and repression, both of which, surprisingly, were exaggerated in PRs.

This study differs methodologically from prior studies of IRG expression in MS. First, we used a prospective design and a defined, validated outcome measure based on an objective MRI criterion [4] that clearly delineates patients with poor clinical outcomes on IFNβ treatment [7]. Second, we quantified IRG expression with a customized cDNA macroarray assay containing 166 IRGs, selected on the basis of previous microarray experiments. This macroarray has been validated for other disease indications such as IFN-α treatment for hepatitis C virus, and is reproducible, sensitive, and quantitative [23]. For focused quantitative studies of a relatively small number of regulated genes such as the IRGs, the macroarray was well-suited to our research questions. Importantly, phlebotomy was performed in a clinical research unit precisely 12 hours after IFN injection, minimizing variability induced by differences in sampling times. Finally, we used a single IFNβ preparation, likely reducing noise in the molecular response measure. The findings can be extrapolated to IFNβ treatment generally because the molecular response to the three approved IFNβ preparations is highly consistent [26]–[30].

The consistent pattern of exaggerated induction or repression of IRGs in patients who responded poorly was observed at the initial IFNβ injection and confirmed at 6 months, indicating that this exaggerated response was a stable phenotype. Over time, circulating leukocyte subsets change with IFNβ therapy, so it was not surprising that the specific genes identified as distinguishing good and poor responders differed at the two time points. This observation leads to a novel hypothesis: patients with MS who exhibit an exaggerated response to exogenous IFNβ have both a poor treatment response and more severe disease. Indeed, patients categorized as poor responders were more likely to have gadolinium enhancing lesions at baseline, and had a higher T2 lesion burden. Thus, patients with poor response to IFNβ injections already had more active disease at treatment start. We believe the explanation for this observation is that the response to exogenous IFNβ injections reflects responses to endogenous type 1 IFN. It has been well recognized that some, but not all, MS patients experience relapses during viral infection, which strongly induces endogenous type 1 IFN. An exaggerated response to type 1 IFN plus disease driven by aberrant innate immunity might explain the response to viral infection in a subset of MS patients. Our hypothesis would predict that patients experiencing disease activation with viral infections would have more severe disease. This has not been studied.

Our study has several limitations. First, there was no placebo-treated, or untreated comparison group, so the subset we’ve identified could simply represent more severe disease, and the outcome might have nothing to do with IFNβ treatment. Second, advances in microarray technology during the course of this longitudinal study now permit more quantitative assessment of the RNA we collected. Those experiments, currently ongoing, have the potential to confirm or refute the current findings, and possibly to extend them considerably. Third, the classification of poor treatment response was based entirely on a prospectively defined MRI outcome. The clinical outcome for poor and good response subgroups needs to be established in long-term follow-up studies, which are ongoing. Lastly, our findings must be confirmed in separate patient cohorts and by independent investigators.

Our hypothesis is consistent with a recent report by Axtell and colleagues suggesting that fundamentally different pathogenic pathways in MS subjects underlie the differential response to IFNβ therapy [12]. They reported that IFNβ was effective in suppressing murine experimental allergic encephalomyelitis caused by transfer of T_H_1 cells, but exacerbated disease caused by transfer of T_H_17 cells. Intriguingly, two IRGs we found to be markedly regulated by IFNβ (IL2, PI3K) would be predicted to act in a manner consistent with the hypothesis proposed by Axtell et al. Specifically, we observed exaggerated inhibition of IL2 and exaggerated induction of PI3K, which together could cause T_H_17-mediated inflammation that would be resistant to inhibition by gene products of the type I IFN response, including IL10 [31], [32]. van Baarsen et al. [33] found that high expression of 15 IRGs before starting IFNβ treatment predicted limited induction of IRGs after treatment. They concluded that the ability to respond to IFNβ pharmacologically was intrinsic to circulating blood cells, before the introduction of IFNβ. Our results are in part consistent with Comabella et al., [34] who found overexpression of IFN-induced genes prior to IFNβ treatment in patients with a poor clinical response. Our findings also generally agree with recent genome-wide association studies showing that MS susceptibility was associated with a SNP near the IRF8 gene [35] and another SNP within the gene encoding TYK2 [36], a cytoplasmic tyrosine kinase required for IFN responses. The IRF8 susceptibility SNP was associated with increased expression of a wide variety of IFN pathway genes, and the TYK2 SNP encoded a variant amino acid in the kinase domain that was predicted to modify IFN-driven gene expression. These observations suggest that the type I IFN pathway, a critical component of the innate immune system, may play a pathogenic role in some patients with MS. Finally, our results are reminiscent of findings related to the IFN-treatment response in patients with chronic hepatitis C infection [37]. Patients with a poor treatment response to pegylated IFNα and ribavirin had high levels of IRG products in pre-treatment liver biopsies compared to patients with a good response, suggesting that activation of the endogenous IFN system was not only ineffective in clearing hepatitis C virus, but was also predictive of a poor response to exogenous IFN therapy.

Despite these recent reports, predictive biomarkers for IFNβ treatment failure have not been identified or validated [6], [14], [16], [38]–[48]. Our observations introduce the novel concept that the differential treatment response to IFNβ is not explained by a specific set of IRGs, but rather by a generally augmented IRG response to IFNβ injections, and that response to IFNβ injection unmasks a subset of patients who not only respond poorly to IFNβ treatment, but who have a pathogenetically distinct form of MS. This hypothesis has at least two major implications. First, because the response to an initial IFNβ injection strongly correlated to response to therapy 6 months later it should be possible to develop assays that identify patients who will have a poor response to IFNβ therapy, enabling the tailoring of disease-modifying therapy for individual patients. It may be possible to develop an assay that could be applied to decide whether a patient is likely to do poorly on IFNβ therapy. Because MS lesions can irreversibly injure axons [49], optimizing therapy quickly might substantially benefit patients. Development and validation of a biomarker based on these observations, is beyond the scope of this initial report, however.

A more wide-ranging implication relates to MS pathogenesis. Mechanistic proposals for disease pathogenesis have focused on adaptive immunity, particularly immune responses directed against myelin constituents. We found that IFNβ recipients who became PRs already had higher levels of disease activity and disease burden, as measured by MRI, upon entering the study. We hypothesize that these patients exhibit a pathogenic pathway *that is characterized in part by their response to endogenous type 1 interferon*. A corollary is that differences in innate immunity, either within type I IFN pathways or affecting the expression levels of IRGs indirectly, are determinant for enhanced disease severity in PRs.

In that regard, IFNβ injection can be viewed as a provocative test that may expose a genetically or epigenetically determined type 1 IFN response that contributes to pathogenesis. In these individuals, exogenous IFNβ elicits an exaggerated response, either by upregulation from a lower pre-injection IRG expression level^32^ or through augmented responses from an equivalent pre-injection baseline. In either case, the findings in this study implicate innate immunity in MS pathogenesis from its onset and may provide novel insights into the fundamental disease process, along with new therapeutic targets and hope for personalized use of IFNβ.

## Supporting Information

Figure S1A **Scatter plots showing the IFNβ** **molecular response at baseline and 6 months for 57 of 85 patients.** The study subject number is indicated above each plot; response at baseline is shown on the x-axis and at 6 months is shown on the y-axis. For each subject, the induction ratio for each of 166 genes is shown at the two time points. The variability of the molecular response between the two time points is indicated by deviation from the diagonal line in each plot. A consistent response at the two time points is evident except for patients 7, 21, and 25 (see text). Also, response consistency was similar between patients with poor treatment response (circled study subject numbers).
(EPS)

Figure S1B **Scatter plots showing the IFNβ** **molecular response at baseline and 6 months for the remaining 37 of the 85 patients.** The study subject number is indicated above each plot; response at baseline is shown on the x-axis and at 6 months is shown on the y-axis. For each subject, the induction ratio for each of 166 genes is shown at the two time points. The variability of the molecular response between the two time points is indicated by deviation from the diagonal line in each plot. Response consistency was similar between patients with poor treatment response (circled study subject numbers).
(EPS)

Figure S2 **Scatter plots for 10 individual patients showing a consistent response over 24 months.** Five good and 5 poor responders were randomly selected from each group with macroarray data at baseline, 6 months, and 24 months. The first 3 columns show patients with a good treatment response, and the last 3 columns are patients with a poor treatment response. Columns 1 and 4 compare responses at baseline and 6 months; columns 2 and 5 compare responses at 6 and 24 months; and columns 3 and 6 compare responses at baseline at 24 months. Note the consistency of response for all time points and that the molecular response is consistent regardless of whether treatment response is good or poor.
(EPS)

Table S1 (DOC)
