# Supplementary material for: Excessive Biologic Response to IFNβ Is Associated with Poor Treatment Response in Patients with Multiple Sclerosis
Source: PLoS One. 2011 May 13;6(5):e19262. doi: 10.1371/journal.pone.0019262 (PMC3094352; doi:10.1371/journal.pone.0019262)
Supplement: Table S1 — (DOC) [file pone.0019262.s004.doc]

**Supplemental Table 1.** Names and GenBank accession numbers for the 166 type 1 interferon-responsive genes selected for the customized macroarray

| **Gene** | **Accession No.** |  | **Gene** | **Accession No.** |  | **Gene** | **Accession No.** |  | **Gene** | **Accession No.** |
| --- | --- | --- | --- | --- | --- | --- | --- | --- | --- | --- |
| **2-5OAS** | NM_002534 |  | **G1P3** | NM_002038 |  | **IP-10** | X02530 |  | **PDK2** | NM_002611 |
| **a1-AT** | K01396 |  | **Gadd45** | M60974 |  | **IRF4** | U52682 |  | **PGK** | V00572 |
| **ADAM17** | U69611 |  | **GATA 3** | X58072 |  | **IRF1** | L05072 |  | **PI3K** | NM_006219 |
| **Adaptin** | AF068706 |  | **GBP2** | M55543 |  | **IRF2** | X15949 |  | **PIAS** | AF077954 |
| **Akt-1** | NM_005163 |  | **Gran B** | M17016 |  | **IRF7** | U73036 |  | **PIAS1** | AF077951 |
| **Akt-2** | M77198 |  | **HLADP** | M83664 |  | **ISG15-L** | M13755 |  | **Pig7** | AF010312 |
| **APOL3** | AA971543 |  | **HLADRA** | J00194 |  | **ISG20** | NM_002201 |  | **PKR** | NM_002759 |
| **ATF 2** | X15875 |  | **HLAE** | X56841 |  | **ISGF3g** | M87503 |  | **plectin** | U53204 |
| **Bad** | U66879 |  | **Hou** | U32849 |  | **JUN** | J04111 |  | **PLSCR1** | AF098642 |
| **Bax** | U19559 |  | **HPAST** | AF00144 |  | **L1CAM** | M74387 |  | **PSMB9** | X66401 |
| **Bcl-2** | M14745 |  | **Hsf1** | M64673 |  | **L-Selectin** | M25280 |  | **Raf** | X03484 |
| **BST2** | D28137 |  | **Hsp90** | X15183 |  | **MAP2K3** | NM_002756 |  | **RCNI** | D42073 |
| **C1-INH** | NM_000062 |  | **IDO** | NM_002164 |  | **MAP2K4** | L36870 |  | **RGS2** | NM_002923 |
| **C1orf29** | NM_005951 |  | **IFI16** | M63838 |  | **MAP3K11** | NM_002419 |  | **RHO GDP** | L20688 |
| **C1r** | NM_001733 |  | **IFI-17** | J04164 |  | **MAP3K14** | NM_003954 |  | **Ribonuc** | NM_003141 |
| **C1S** | J04080 |  | **IFI35** | U72882 |  | **MAP3K3** | U78876 |  | **RIG-1** | AF038963 |
| **Caspase 1** | M87507 |  | **IFI44** | D28915 |  | **MAP3K4** | NM_005922 |  | **SERPIN** | NM_000295 |
| **Caspase 7** | U67319 |  | **IFN-44** | D28915 |  | **MAP3K7** | NM_003188 |  | **Smad1** | U59423 |
| **Caspase 9** | U60521 |  | **IFI60** | AF083470 |  | **MAP4K1** | NM_007181 |  | **SNN** | NM_003498 |
| **CBFA** | NM_004349 |  | **IFIT1** | M24594 |  | **MAPK13** | AF004709 |  | **SOCS-1** | N91935 |
| **CCR1** | L09230 |  | **IFIT2** | NM_001547 |  | **MAPK7** | NM_002749 |  | **SOCS2** | AF020590 |
| **CCR5** | U54994 |  | **IFIT4** | NM_001549 |  | **Met-onco** | NM_000245 |  | **SSA1** | NM_003141 |
| **CD14** | NM_000591 |  | **IFIT5** | NM_012420 |  | **MIP-1b** | NM_002984 |  | **STAT1** | M97935 |
| **CD3e** | NM_012099 |  | **IFITM2** | NM_006435 |  | **MMP-1** | M13509 |  | **STAT2** | M97934 |
| **CEACAM** | NM_001712 |  | **IFITM3** | X57352 |  | **MMP-9** | NM_004994 |  | **STAT4** | L78440 |
| **c-fos** | NM_005252 |  | **IFN-17** | M13755 |  | **MT1H** | NM_005951 |  | **STAT5A** | L41142 |
| **c-myc** | L00058 |  | **IFN-9/27** | J04164 |  | **MT1X** | NM_005952 |  | **TAP1** | X57522 |
| **Collagen** | J03464 |  | **IFNAR1** | J03171 |  | **MT2A** | NM_005953 |  | **TFEC** | NM_012252 |
| **COMT** | M58525 |  | **IFNAR2** | L42243 |  | **MX1** | M33882 |  | **TGFbR2** | D50683 |
| **CREB** | NM_004379 |  | **IFNGR1** | J03143 |  | **MX2** | M30818 |  | **TGFbR3** | L07594 |
| **CXCL11** | NM_005409 |  | **IFNGR2** | U05875 |  | **NF-IL-6** | X52560 |  | **TIMP-1** | M59906 |
| **CXCR4** | AF005058 |  | **IkBa** | M69043 |  | **NFkB** | M58603 |  | **TNF-a** | X01394 |
| **CYB56** | NM_007022 |  | **IL15** | U14407 |  | **NMI** | Y00664 |  | **TNFAIP6** | NM_007115 |
| **Cyp19** | M28420 |  | **IL18 BP** | AB019504 |  | **NT5e** | X55740 |  | **TOR1B** | NM_014506 |
| **DDX17** | U59321 |  | **IL1RN** | NM_000577 |  | **OASL** | NM_003733 |  | **TRAIL** | U37518 |
| **Def-a3** | NM_005217 |  | **IL2** | NM_000586 |  | **P4HA1** | M24486 |  | **UBE2L6** | NM_004223 |
| **Destrin** | S65738 |  | **IL2Rg** | NM_000206 |  | **p53** | M14694 |  | **USP18** | NM_017414 |
| **Elastase 2** | M34379 |  | **IL6** | X04602 |  | **p57Kip2** | U22398 |  | **VegFC** | U43142 |
| **F-actin** | U56637 |  | **IL8Rb** | NM_001557 |  | **p70 K** | M60724 |  | **Viperin** | AF026941 |
| **Fas-L** | U08137 |  | **iNOS** | U20141 |  | **PAI-1** | M16006 |  | **WARS** | X62570 |
| **FK506** | AF038847 |  | **Int-6** | U62962 |  | **PDGF-a** | X06374 |  |  |  |
| **FLJ20035** | AK000042 |  | **integ-b-6** | NM_000888 |  | **PDK1** | Y15056 |  |  |  |
